# Research on Task Complexity Measurements in Human—Computer Interaction in Nuclear Power Plant DCS Systems Based on Emergency Operating Procedures

**DOI:** 10.3390/e27060600

**Published:** 2025-06-04

**Authors:** Ensheng Pang, Licao Dai

**Affiliations:** 1 School of Nuclear Science and Technology, University of South China, Hengyang 421001, China; 20222010110747@stu.usc.edu.cn; 2 Institute of Human Factors, University of South China, Hengyang 421001, China

**Keywords:** nuclear energy, nuclear power plant, nuclear safety, human–computer interaction, task complexity

## Abstract

Within the scope of digital transformation in nuclear power plants (NPPs), task complexity in human–computer interaction (HCI) has become a critical factor affecting the safe and stable operation of NPPs. This study systematically reviews and analyzes existing complexity sources and assessment methods and suggests that complexity is primarily driven by core factors such as the quantity of, variety of, and relationships between elements. By innovatively introducing Halstead’s *E* measure, this study constructs a quantitative model of dynamic task execution complexity (TEC), addressing the limitations of traditional entropy-based metrics in analyzing interactive processes. By combining entropy metrics and the *E* measure, a task complexity quantification framework is established, encompassing both the task execution and intrinsic dimensions. Specifically, Halstead’s *E* measure focuses on analyzing operators and operands, defining interaction symbols between humans and interfaces to quantify task execution complexity (TEC). Entropy metrics, on the other hand, measure task logical complexity (TLC), task scale complexity (TSC), and task information complexity (TIC) based on the intrinsic structure and scale of tasks. Finally, the weighted Euclidean norm of these four factors determines the task complexity (TC) of each step. Taking the emergency operating procedures (EOP) for a small-break loss-of-coolant accident (SLOCA) in an NPP as an example, the entropy and *E* metrics are used to calculate the task complexity of each step, followed by experimental validation using NASA-TLX task load scores and step execution time for regression analysis. The results show that task complexity is significantly positively correlated with NASA-TLX subjective scores and task execution time, with the determination coefficients reaching 0.679 and 0.785, respectively. This indicates that the complexity metrics have high explanatory power, showing that the complexity quantification model is effective and has certain application value in improving human–computer interfaces and emergency procedures.

## 1. Introduction

With the accelerated digital transformation of nuclear power plants, digital control systems (DCS) have enhanced monitoring efficiency and also introduced new challenges to human–computer interaction (HCI) task complexity. Studies have indicated [1,2,3,4,5] that in emergency situations, operators are required to quickly perform critical operations through multi-layer menus, complex logical procedures, and information-dense interfaces. At such times, issues like the keyhole effect in interface information display and the distraction caused by switching between primary and secondary tasks can significantly reduce the speed and accuracy of operators’ decision-making, thereby directly affecting the safe operation of nuclear power plants. To address this issue, it is necessary to scientifically quantify the complexity of tasks involving human–computer interaction. By analyzing the actual workflow of operators, the difficulty of complex operations can be transformed into a measurable indicator that can clearly identify bottlenecks in interface design or operational procedures. For instance, improvements such as reducing unnecessary menu levels, optimizing information layout, and simplifying logical branches all need to be based on the results of complexity quantification. This research approach of problem identification–optimized design effect verification can provide important support for the human–computer collaborative safety efforts in digitalized nuclear power plants.

The essence of task complexity stems from the dynamic coupling of multi-dimensional factors. As Xing [6,7] described, complexity is constituted by the quantity of, variety of, and relationships between basic elements. Similarly, Bedeny [8] defined task complexity as an intrinsic characteristic that is determined by the amount of uncertainty and variety and the number of task elements’ coupling relationships. When mapped to nuclear power plant (NPP) HCI scenarios, these complexity dimensions manifest as hierarchical task structures, the quantity and diversity of interactive components, network topologies of logical relationships, and combinations of information volume and types, collectively forming the complexity landscape of HCI tasks.

Traditional evaluations of operating procedures and human–computer interfaces primarily rely on qualitative assessments. For example, the *Human–Computer Interface Design Review Guide*, developed by the U.S. Nuclear Regulatory Commission (NRC) [9], and the checklist for evaluating emergency procedures in nuclear power plants [10] are both qualitative tools. Although qualitative assessments can optimize user experience, they fail to identify the intrinsic indicators that affect usability. This highlights the need for quantifying complexity to assess the intrinsic factors influencing human–computer interface interactions and the quality of emergency operating procedures. Existing methods for quantifying complexity mainly follow the development paths of information theory and software science. One well-known method is McCabe’s cyclomatic complexity [11], which measures program complexity based on the number of linearly independent paths in a program’s control flow graph. However, this method is limited: it focuses only on the structural complexity of the program, ignoring its size. In contrast, Halstead’s *E* measure [12] considers the program’s size but overlooks the structural impacts. Park et al. [13,14,15,16] proposed a complexity measurement method for emergency procedures based on entropy theory. This method categorizes logical nodes in a program to calculate entropy values. However, using entropy theory to measure the complexity of human–computer interface interactions is challenging and often meaningless. From the perspective of interface interaction, the most significant factor affecting operational efficiency is the scale of operations. A well-designed human–computer interface should achieve operational goals with minimal steps. Therefore, the complexity of human–computer interface interactions is more aligned with Halstead’s *E* measure. This measure evaluates complexity from the perspectives of quantity and variety, considering operators and operands, as well as the types and frequencies of elements to assess scale and workload. Both entropy and *E* measures have been demonstrated to be useful for measuring task complexity. For instance, Xu et al. [17] validated the impact of different presentation styles of emergency operating procedures on personnel performance based on Park’s entropy measure. Zhang et al. [18] further extended entropy measures to aerospace tasks, verifying their correlation with operational time. Nieminen [19] used *E* measures and the WOOD task complexity measurement method to calculate the complexity of mobile application UI interaction tasks.

In recent years, research on task complexity measurements has expanded into other high-risk fields. For instance, in the aerospace field, Tamaskar [20] proposed a framework for measuring the complexity of aerospace systems, emphasizing modularity and coupling, which is similar to the static complexity indicators in our study. Moreover, Liu [21] developed a pilot workload measurement model based on task complexity, which is directly related to our use of NASA-TLX to evaluate subjective workload. These studies provide an important theoretical basis and methodological reference for our research.

**Figure 1:**
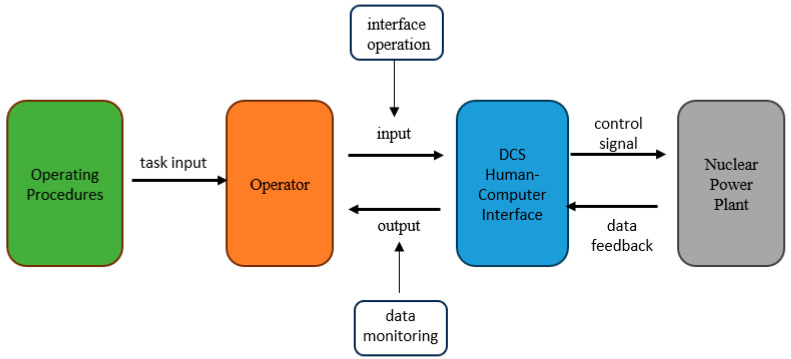
Nuclear power plant DCS human–computer interaction framework.

In light of the above discussion, this study proposes a dual-perspective framework for quantifying task complexity, ranging from the intrinsic nature of the task to its execution. Building on Park’s entropy metrics [13], Halstead’s *E* measure is innovatively integrated to quantify task complexity in NPP DCS HCI from both static task inputs and dynamic task outputs. As shown in Figure 1, the framework evaluates task complexity from two perspectives: task ontology (based on procedures) and task execution (based on interface interactions). First, complexity is defined relative to human operators, as Xing [7] emphasized: complexity has an observer effect. The key to assessing interface complexity lies in whether it supports operators in achieving task goals efficiently. For instance, experienced operators are more familiar with the interface and emergency procedures; they can quickly identify key parameters and emergency handling pathways. Different individuals have varying perceptions of the same human–computer interface. Therefore, we believe that when measuring the complexity of human–computer interface interaction tasks, the layout of the interface should be taken into account. We have always held this view: if the interface effectively supports personnel in completing tasks, it indicates that the interface design is adequate. The key factor affecting personnel efficiency lies in the scale of interaction with the interface. This is because the larger the scale, the longer the operation time may be, and the more intense the cognitive resource competition for the group tasks.

Assuming a small-break loss-of-coolant accident (SLOCA) in the primary loop, Event-Based Emergency Operating Procedures (EOP) guide operators to interact with the DCS system. The DCS human–computer interface serves as the direct communication channel between operators and the system, receiving commands and providing feedback. Simultaneously, the DCS interacts with other plant components via controllers and sensors. Operators play a critical role in translating static procedures into dynamic actions. To reduce operator workload and enhance efficiency, task comprehensibility and interface usability are paramount. This study proposes a method that combines entropy and Halstead’s *E* measure to quantify task complexity and interface operation difficulty, providing theoretical support for optimizing the DCS’s interface design and EOPs. It is worth noting that this study focuses on the single-role interaction between individual operators and digital control systems. Due to the limitations of implementation conditions, we do not consider the interactive effects between people within teams. The research framework is limited to the task entity and interface operations.

## 2. Methodology

Task complexity quantification is key to understanding the potential risks in HCI. This study proposes a multi-dimensional framework that considers element quantity, variety, and relationships. Halstead’s *E* measure and entropy theory are integrated to evaluate complexity from task execution and intrinsic logic dimensions. Specifically, task complexity is decomposed into four core metrics as follows:

(1) Task Execution Complexity (TEC): This quantifies the cognitive and operational load of the dynamic interactions between the operator and the interface. It is based on Halstead’s E measure, which statistically counts the types and usage frequencies of operators and operands to reflect the redundancy of interaction paths and the density of operational steps.
(2) Task Logical Complexity (TLC): This measures the diversity of logical branches in the task process. It calculates the equivalent class distribution of nodes in the control flow graph of operational steps using first-order entropy. A higher entropy value indicates greater logical complexity.
(3) Task Information Complexity (TIC): This evaluates the quantity and scale of information required for task execution. It is based on the second-order entropy calculation of the information structure diagram. A higher entropy value indicates greater information complexity.
(4) Task Scale Complexity (TSC): This describes the volume characteristics of the task itself. It quantifies the number of operational steps and their logical dependencies using second-order entropy. A higher entropy value indicates a larger scale.

**Figure 2:**
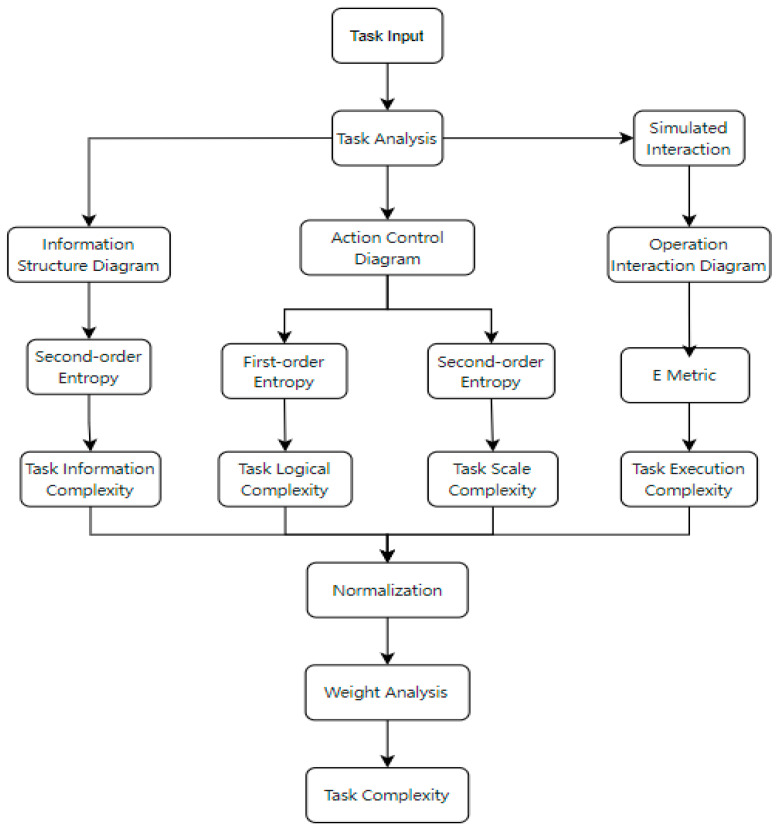
Task complexity calculation process in human–computer interactions.

Finally, a weighted Euclidean norm integrates these metrics into a comprehensive task complexity (TC) value. The calculation process is illustrated in Figure 2.

### 2.1. E Measurement

Halstead’s *E* measurement is used to calculate program complexity, measuring the “effort” or workload required by programmers during development by considering the types and frequencies of operators and operands. Program complexity is proportional to the number of distinct operators and operands that programmers need to distinguish; the more operators and operands there are, the greater the difficulty in understanding and maintaining the program. We define the complexity generated by interface operations as the complexity of actions users perform on the interface to achieve task goals, termed task execution complexity (TEC), and introduce the *E* measurement to quantify this complexity. The calculation formula is shown in Equation (1):

(1) $$E=\frac{\eta_{1}N_{2}\left(N_{1}+N_{2}\right)}{2\eta_{2}}\log_{2}\left(\eta_{1}+\eta_{2}\right)$$

where $\eta_{1}$: represents the number of unique operators, i.e., operators refer to the interactive actions executed by users to achieve task goals. For example, in a DCS interface, operators include clicks, such as clicking on a valve icon to open an operation window; double-clicks, such as double-clicking on an input box to activate data entry; and long presses, such as holding down a reset button to execute an equipment reset. $\eta_{2}$: represents the number of unique operands, i.e., operands refer to the functional components or information units on the interface that need to be operated. For example, operands include the interface elements corresponding to physical devices, such as the cooling water valve VP001 and the main pump 001PO, as well as logical components, such as input boxes and navigation icons. $N_{1}$: is the total frequency of the operators. $N_{2}$: is the total frequency of the operands.

Thus, the *E* measurement reflects the workload during task execution; the more complex the interface interaction logic and the more operators and operands there are, the higher the task load. Park [13] argues that the *E* measurement is an absolute complexity measurement method. Although using consistent operators and operands is challenging, it is highly effective in analyzing interface task complexity, as operators and operands in interfaces are typically explicitly defined by designers. Chewar [22] compared Halstead’s *E* measurement and McCabe’s measurement in evaluating software psychological complexity, finding that the *E* measurement effectively calculates the number of psychological discriminations in software maintenance. Nieminen [19] demonstrated the effectiveness of the *E* measurement in measuring interface task complexity by combining the *E* measurement with WOOD’s task complexity calculation using data from 1460 UI interfaces.

**Figure 3:**
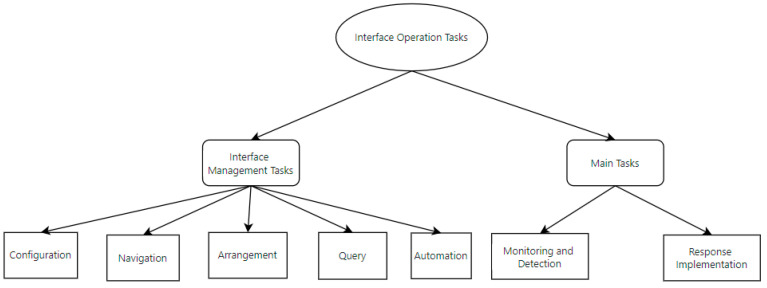
Classification of interface operation tasks.

In terms of interface operation tasks, we define them as the process by which operators interact with the interface to achieve various task objectives while performing complex tasks. As shown in Figure 3, interface operation tasks are divided into two categories: interface management tasks and main tasks. Interface management tasks are auxiliary tasks that help operators efficiently manage the interface to support the execution of main tasks, such as adjusting window layouts and navigating menus. These tasks do not directly achieve the goals but facilitate information retrieval and operation. Main tasks are task types that directly achieve nuclear safety goals through interface interaction. Based on O’Hara’s framework [4], main tasks usually include the following four sub-goals: situation assessment, monitoring and detection, response planning, and response implementation. However, this study focuses on the task types that directly interact with the interface, namely, monitoring and detection: obtaining system status information through continuous interaction with the interface, such as real-time monitoring of the reactor containment–pressure curves and checking valve-open/close statuses; and response implementation: executing specific commands through interface operations, such as clicking on a safety valve icon and entering an opening value to relieve pressure. Situation assessment and response planning primarily engage the operator’s working memory, experience, or team discussions and involve internal cognitive activities rather than direct interface interaction. Therefore, they are not included in the “Main Tasks” category in Figure 3. This definition ensures that the complexity quantification model focuses on observable and recordable interface interaction behaviors, thus aligning with the experimental design based on operation logs and execution time in Section 4. In this study, operators first clarify the task objectives through operating procedures, i.e., the main task objectives to be accomplished. To achieve these objectives, they then perform a series of interface management tasks to support the execution of the main tasks, ultimately completing response implementation or monitoring and detection tasks to achieve the overall task goals. In the task analysis framework of this study, the Hierarchical Task Analysis (HTA) method was adopted to analyze and model tasks in depth, with specific details elaborated in Section 3.

Within the framework of defining interface operation tasks and based on the actual human–computer interaction process, we extend the definitions of operators and operands. In interface interactions, users typically need to perform multiple interactive actions such as information retrieval, navigation display, and window operations. For example, when assessing the pressure and temperature of containment, operators need to review relevant data to decide whether to activate the containment spray system. Here, the operator navigates the interface to locate the window displaying the relevant information and retrieves the required information through interactive actions like clicking. We define this information retrieval action, specifically performed to complete the main task, as an operator. Similarly, when an operator uses a spray valve for pressure reduction, they first need to open the spray valve operation window and enter the desired flow values in the input boxes. In this process, the typing action is defined as an interactive operator, while the keyboard serves as the operand for this interactive action. The interactive components, actions, and their corresponding functional descriptions involved in the actual operation process are detailed in Table 1. These elements will inform the modeling of interaction diagrams in the application examples in Section 3.

Note that, in order to enhance the consistency and representativeness of the classification, we have conducted the classification process by referring to the functional types and perceptual style differences of the interface elements. For example, all valve operations adopt a unified style and thus are classified as a single operand type. In contrast, controllers, which have significant differences in style and perception, are respectively categorized into multiple operand types, such as VB (borated water valve), VP (cooling water valve), and VN; these valve components share the same style and operation mode. Considering this, they do not significantly consume the operator’s cognitive resources. Therefore, in Table 1, all valve components are classified as the same type of operand. Similarly, although the controllers share the same interaction mode, their styles vary significantly, increasing perceptual complexity and requiring the operators to expend cognitive resources when performing tasks. Thus, in Table 1, the three controller styles are categorized into three separate classes. Although we have provided clear definitions and standardized annotations for operators and operands, there may still be slight differences in semantic interpretation in some boundary cases, which may cause minor deviations in the E value. However, this will not affect the identification of the regression trend between variables. This classification method facilitates the construction of interaction diagrams and the calculation of the E metric.

**Figure 4:**
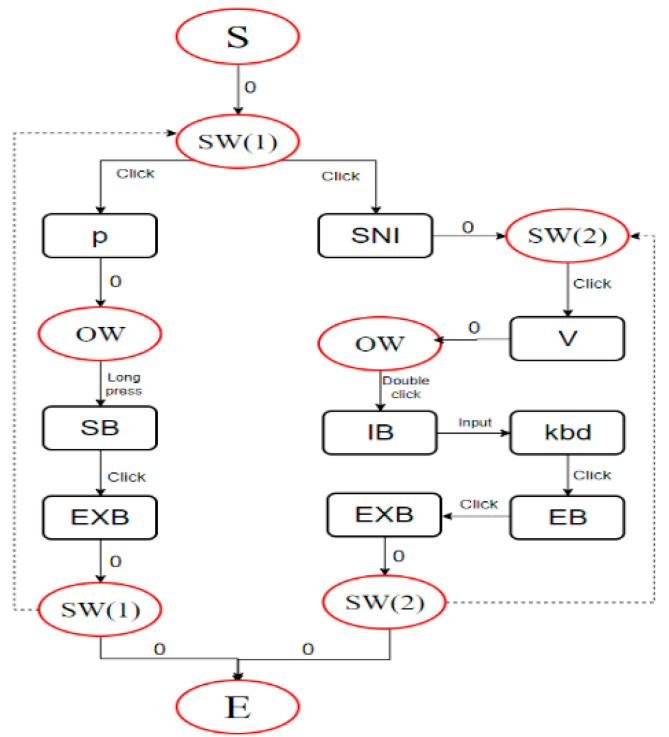
Interaction diagram example.

Below is a simple example of the E-metric calculation. Suppose we operate a valve for flow control and to start a pump. The interaction behavior for completing this task is shown in Figure 4. The nodes in the graph represent interactive components on the interface, i.e., operands in the E metric, while the edges represent interactive actions with the components, i.e., operators in the E metric. The direction of the edges indicates the flow of operations. The elliptical nodes represent interfaces or operation windows, which, along with the start and end nodes, are not included in the calculation but are essential for connecting operations. The dashed lines indicate that after completing a series of actions, the system screen is not covered by a new screen, and the next series of operations starts from this system screen. The interaction diagram visually shows the interaction flow, allowing for quick statistics of the number of operators and operands, and the calculation yields TEC = E ≈ 145.19.

### 2.2. Entropy Measurement

Nuclear power plants utilize accident procedures to guide operators in accident handling [23]. While experienced operators can execute routine tasks without procedural guidance [24], even moderately complex tasks like normal reactor startups and shutdowns, most operators report a significant cognitive load [25]. Therefore, in emergencies, operators must rely on procedures. The comprehensibility of procedures is crucial for operators to complete tasks successfully. Current nuclear power plant accident procedures are mainly divided into event-oriented EOP procedures and Symptom-Based Operating Procedures (SOPs). Although EOP and SOP procedures differ, their basic logical structures are similar, consisting of basic If-Then logic structures. At each decision point, operators make yes or no decisions based on the plant’s conditions, thereby determining the procedure’s direction. However, SOP procedures have more reasoning and decision points compared to EOP procedures. This study uses EOP emergency operating procedures for task complexity analysis.

Boring pointed out that procedure-based manual control actions may be delayed due to the complexity of procedure steps and other factors, and the amount of information that operators need to attend to is a major factor affecting the usability of procedures. Long [26] proposed that the amount of information required to execute steps is a key factor affecting procedure usability. Similarly, Macwan [27] and Peng [28] pointed out that the logical structure and step size of procedures are the main factors contributing to procedure complexity. Building on these findings, we use the concept of entropy to measure task complexity based on emergency operating procedures. The entropy concept was originally introduced by physicist Clausius to describe the degree of disorder or chaos in a system. Later, Shannon further developed the concept in information theory, where Shannon entropy [29] is used to measure the average information content of an information source. A higher entropy indicates greater uncertainty, while a lower entropy indicates more predictability. Shannon provided the mathematical definition of entropy in Equation (2):

(2) $$H=-\sum_{i=1}^{N}P\left(A_{i}\right)\log_{2}P\left(A_{i}\right)$$

where

- H represents information entropy.
- N is the number of information sources.
- $A_{i}$ represents the ii-th information source.
- $P\left(A_{i}\right)$ represents the probability of the ii-th information source occurring.

The concept of entropy has been applied in various fields. In software engineering, entropy has been used to evaluate software complexity. Davis [30], Lew [31], and others have validated the applicability of entropy as a complexity metric and provided a theoretical foundation for quantifying complexity. Zhang [18] used entropy theory to measure the operational complexity of spaceflight and experimentally validated the effectiveness of this measure. Mowshowitz [32] proposed a graph complexity measurement method based on entropy. The author introduced two types of entropy for measuring graph complexity: first-order entropy and second-order entropy.

**Figure 5:**
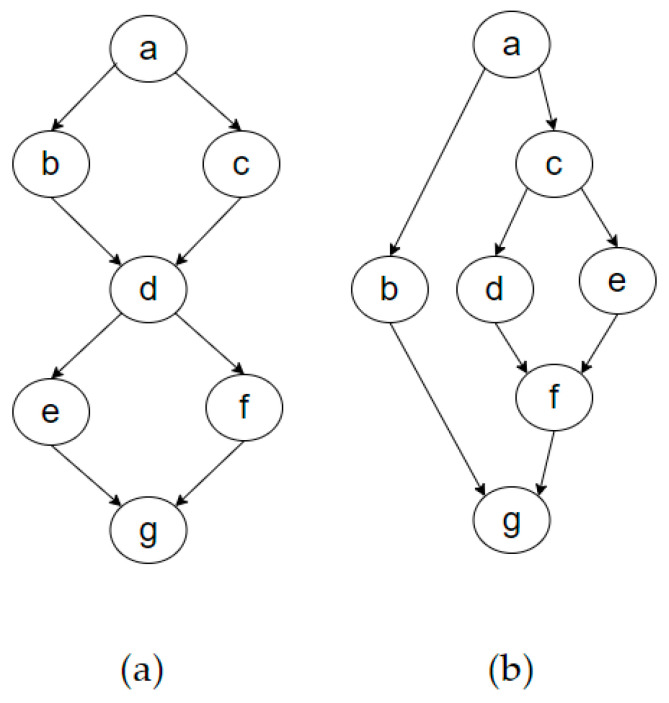
Example diagram of graph entropy calculation.

To explain the characteristics and calculation methods of first-order and second-order entropy, we use Davis’s software complexity measure [30] as an example, as shown in Figure 5. This figure illustrates two program control graphs. First, we calculate the first-order entropy by classifying nodes based on their in-degree and out-degree. If multiple nodes have the same in-degree and out-degree, they can be grouped into the same equivalence class. Graphs containing many nodes within the same equivalence class generally exhibit lower entropy values. This indicates that when the control logic has a certain regularity, the program’s context is easier to understand, and this regularity is quantified by first-order entropy. Based on this classification method, Table 2 identifies the equivalence classes for graphs Figure 5a,b.

As can be seen from Table 2, the nodes in Figure 5a can be divided into four categories, and the probability of each category of nodes can be obtained. For example, the probabilities of the Type-I nodes and the Type-II nodes are 1/6 and 4/6, respectively. Then, by substituting N = 4 and P(Ai) into Formula (2), the first-order entropy of Figure 5a can be calculated.

$$H_{a_{1}}^{\prime }=-\sum_{i=1}^{4}P\left(A_{i}\right)\log_{2}P\left(A_{i}\right)=1.664$$

Similarly, calculate $H_{a_{2}}^{\prime }$ = 2.128. As expected, the value of $H_{a_{2}}^{\prime }$ is greater than that of $H_{a_{1}}^{\prime }$ because Figure 5a is more regular and has more regularity than Figure 5b. The first-order entropy is mainly used to measure the simple diversity in the system. The higher the diversity, the more complex the classification of node types, and the higher the first-order entropy value.

The calculation method for second-order entropy is similar. Second-order entropy is based on the neighborhood characteristics of nodes, taking into account the properties of their one-hop neighbors. If two nodes have the same neighboring nodes within one hop, they are considered to belong to the same class. This classification approach is more suitable for analyzing the global complexity of systems, especially in graphs or networks with nested relationships or complex interactions. Additionally, as the size of the graph increases, the number of classes also increases, as the structural complexity of the graph typically becomes more intricate. Thus, second-order entropy further incorporates contextual relationships, representing the amount of information required to understand the graph. The second-order entropy classification for graph (a) is shown in Table 3.

Table 3 shows the classification scheme required for calculating the second-order entropy. The second-order entropies calculated according to this classification scheme are $H_{a_{1}}^{\prime \prime }=2.236$, $H_{a_{2}}^{\prime \prime }=2.521$ The second-order entropy of $a_{2}$ is higher than its first-order entropy because the structure of nodes in $a_{2}$ is more complex. In particular, the nested structure makes the neighbor characteristics of nodes more complex compared to those in $a_{1}$.

**Figure 6:**
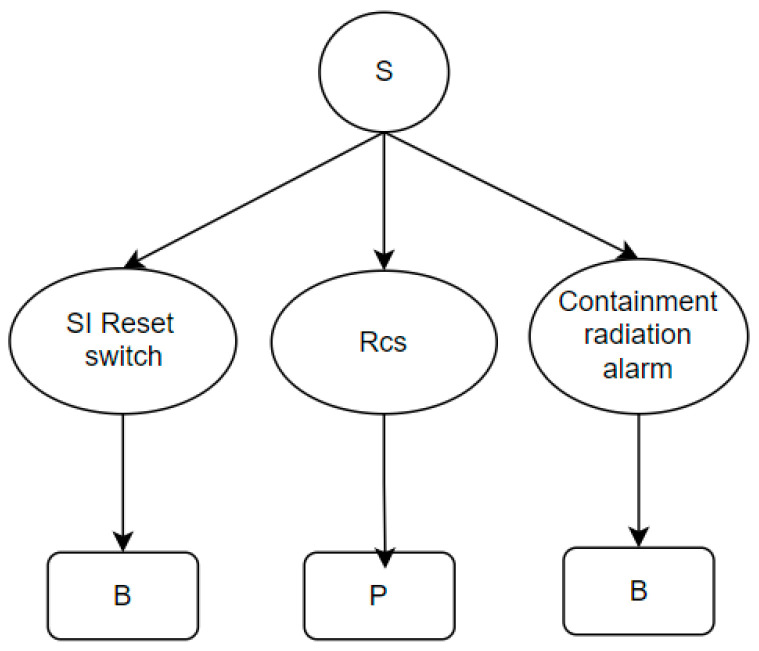
Information structure diagram example.

We use information structure diagrams to measure the information complexity of the operating procedures. The second-order entropy of the information structure diagram is used to quantify its complexity. The reason for using second-order entropy is that its classification method more effectively captures the scale of the graph, thereby measuring the complexity of the information structure diagram. We classify the information types in nuclear power plant operating procedures into control information (for example, switch statuses and alarms) and process variables (for example, temperature and pressure trends). An example of an information structure diagram is shown in Figure 6.

In the information structure diagram, the bottom-level nodes represent information types, such as Boolean values for switch and alarm statuses and continuous variables for reactor temperature and containment pressure. Based on this, second-order entropy is used to calculate the complexity of the graph, thereby measuring the amount of information in the procedure. For example, in Figure 6, the second-order entropy is calculated as H′′ = 2.807.

## 3. Case Study

In this section, we analyze the small-break loss-of-coolant accident (SLOCA) in the primary coolant loop of a nuclear power plant. Based on the accident procedure and interface operations, we calculate the E value and entropy value. We extract step 25 from the SLOCA recovery procedure for detailed calculations. Table 4 shows part of the procedure, and the complete list of task steps is provided in Appendix A.

### 3.1. Hierarchical Task Analysis

In the task analysis framework of this study, Hierarchical Task Analysis is used to systematically deconstruct the emergency operating procedures in the context of a small-break loss-of-coolant accident. HTA helps break down the tasks and sub-goals and clarify the operators’ objectives and information needs at various stages, thereby identifying the critical nodes of cognitive load within the task sequence. Compared with the previous applications of HTA, which were mainly used for developing training manuals and interface process design [33,34], this study further integrates the HTA output with quantitative complexity measures to quantify the operational complexity of each task step. The improvement of this method lies in the fact that HTA is no longer merely used for structured task descriptions but serves as the input basis for quantitative analysis. Specifically, HTA clarifies the behavioral boundaries and information units of each task step, providing fundamental semantic support for the statistics of operands/operators and conditional branch counts in complexity calculations. Furthermore, by applying HTA, the textual task descriptions in the emergency operating procedures are linked with the actual executions on the human–computer interface, ensuring these two dimensions of tasks are no longer disconnected.

**Figure 7:**
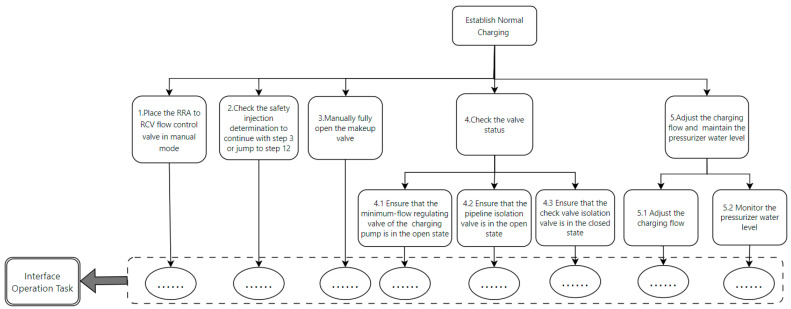
Hierarchical Task Analysis of step 25.

**Figure 8:**
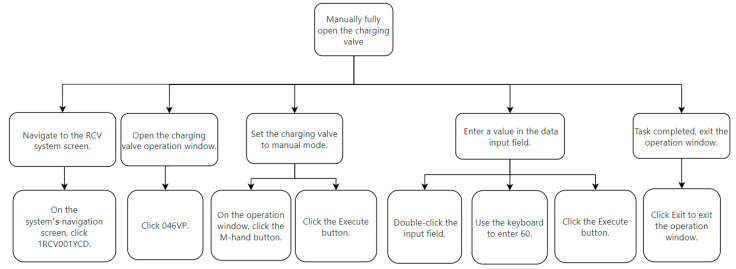
Hierarchical analysis of interface operation tasks.

We decompose the task such that the top level represents the task goal, and the tasks set to achieve the goal are called sub-tasks, which are broken down until they are sufficiently detailed. Taking step 25 as an example, as shown in Figure 7, the top level is the task goal, and the second and third levels are sub-tasks, with the bottom level being interface operation tasks. According to Annett’s research [35], in HTA, the unit of analysis is the operation defined by the goal. These operations are activated by input actions and concluded by feedback. Within the scope of HTA, the goal is defined as the desired system state by humans, the task is the specific method to achieve the goal, and the operation is the behavioral unit executed to achieve the goal. As shown in Figure 7, the task goal is to establish normal feed-water flow, which requires four tasks. Accomplishing these sub-tasks requires operators to interact with the DCS human–computer interface. Interface operation tasks constitute the bottom level of the tasks, and they can be further divided into specific execution goals and operations. For example, step 3 in Figure 7 is analyzed using HTA, as shown in Figure 8.

In the interface operation task shown in Figure 8, the top layer of the figure is the task objective. To achieve this objective, a series of sub-tasks need to be executed, such as navigating to the system window and configuring the operation window. The bottom layer consists of the specific execution to complete these sub-tasks. In this way, we have decomposed a task in the procedure into the smallest components, which helps us to understand the complexity factors in the task process and facilitates the establishment of the operation structure diagram and the interface interaction diagram.

### 3.2. Complexity Measurement

We use the methods described in Section 2 to calculate the complexity, using the E metric to measure task execution complexity and entropy to measure task complexity. Based on step 25 and the task analysis in Section 3.1, we construct the operation structure diagram, information structure diagram, and interface interaction diagram. We then calculate the E value and entropy value based on these diagrams.

**Figure 9:**
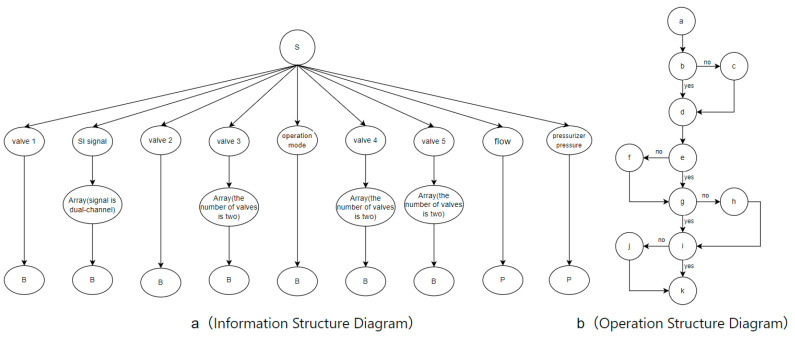
Operation structure and information structure diagrams.

**Figure 10:**
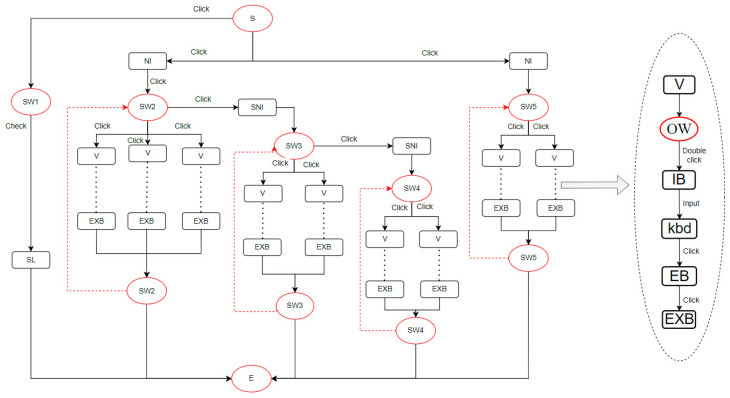
Interaction diagram.

**Figure 11:**
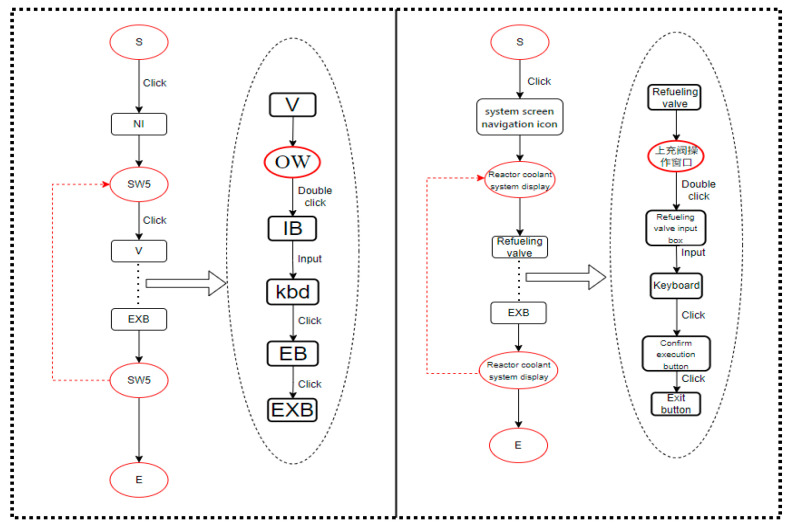
Step 25e, interaction action diagram example.

First, we execute the relevant operations for step 25 on the nuclear power plant DCS simulator, record the interface operations, and analyze the task and instrument information required for execution. We then construct the operation structure diagram and information structure diagram for step 25, as shown in Figure 9, and the interface interaction diagram for step 25, part C, as shown in Figure 10. Take the fifth item of step 2 (the task of adjusting the refueling flow rate) as an example, which is shown in Figure 11 for illustration. In the figure, the oval nodes represent the human–computer interface, and the square nodes represent the interaction objects, such as buttons and the mouse. The edges of the graph represent interaction behaviors, such as clicking and double-clicking. For example, when performing the task of adjusting the refueling flow rate, the operator must first enter the reactor coolant system display and then click the refueling valve button to enter the refueling valve operation window interface. At this time, the operator can double-click the input box in the operation window to enter the corresponding value using the keyboard keys. Finally, by clicking to confirm the execution, the entire task of adjusting the refueling valve flow rate is completed. This diagram clearly displays the operators and operands involved throughout the task. By counting the number and types of operators and operands, the TEC value can be calculated. Finally, we use entropy to calculate the entropy values of the operation structure and information structure diagrams. To construct the information structure diagram, we first analyze the operation information, as shown in Table 5. The operation information is classified into process variables (P), such as changes in the pressurizer water level and primary loop pressure, and Boolean values (B), such as switch and start/stop statuses. Operators must also understand equipment control information, such as the names, quantities, and types of control switches, such as the safety injection reset button, which is classified as type B.

Figure 9a displays the information structure diagram, with the top-level node as the root node, the bottom-level nodes as data types, and the middle layer as component information. The information structure diagram represents the amount of information required to execute each step, and the second-order entropy of the information structure diagram is used to measure the task information complexity. Figure 9b presents the operation structure diagram, which captures the procedural logic. The first-order entropy of this diagram assesses task logical complexity, while the second-order entropy evaluates task scale complexity.

We use second-order entropy to calculate the size of the information structure diagram, which reflects the task information complexity (TIC) of the step. First, we classify the nodes in Figure 9a using the method described in Section 2.2 and substitute the classification results into Equation (2). The calculation result is:

$$S_{TIC}=-\sum_{i=1}^{19}{p\left(A_{i}\right)\log}_{2}p\left(A_{i}\right)=-\left[18(\frac{1}{23}\log_{2}\frac{1}{23})+(\frac{5}{23}\log_{2}\frac{5}{23})\right]=4.019$$

Similarly, we use first-order entropy to calculate the task logical complexity (TLC). We classify the nodes in Figure 9b using the method described in Section 2.2 and substitute the probabilities of different classes into Equation (2). The calculation result is:

$$S_{TLC}=-\sum_{i=1}^{5}{p\left(A_{i}\right)\log}_{2}p\left(A_{i}\right)=-\left[2(\frac{1}{11}\log_{2}\frac{1}{11})+2(\frac{2}{11}\log_{2}\frac{2}{11})+(\frac{4}{11}\log_{2}\frac{4}{11})\right]=2.054$$

We use the second-order entropy calculation method described in Section 2.2 to classify the nodes in Figure 9b and substitute the classification results into Equation (2) to calculate the second-order entropy of Figure 9b. The size of the action control diagram, i.e., the size of Figure 9b, is used to measure the task scale complexity (TSC). The calculation result is:

$$S_{TSC}=-\sum_{i=1}^{11}{p\left(A_{i}\right)\log}_{2}p\left(A_{i}\right)=-\left[11(\frac{1}{11}\log_{2}\frac{1}{11})\right]=3.459$$

From the action control diagram in Figure 10, we count the number of unique operators, unique operands, total operator frequency, and total operand frequency and substitute these values into Equation (1) to calculate the TEC value:

$$S_{TEC}=\frac{\eta_{1}N_{2}\left(N_{1}+N_{2}\right)}{2\eta_{2}}\log_{2}\left(\eta_{1}+\eta_{2}\right)=\frac{5\times 44(45+44)}{2\times 10}\log_{2}\left(5+10\right)=3824.846$$

Based on these values, we use the Euclidean norm to determine the final task complexity (TC) value. The TC value formula is as follows:

$$\begin{array}{c} TC=\sqrt{(\omega_{1}S_{TIC}{)}^{2}+(\omega_{2}S_{TLC}{)}^{2}+(\omega_{3}S_{TSC}{)}^{2}+(\omega_{4}S_{TEC}{)}^{2\;}}\# \end{array}$$

where $\omega_{1}$, $\omega_{2},\omega_{3},\;{and\omega }_{4}$ are the weighting factors. We will use factor analysis to calculate these weights in the following section.

We take the SLOCA accident as an example, selecting 30 steps from the SLOCA procedure as the analysis objects. We calculate the TIC, TLC, and TSC for each step and simulate the operations on the DCS simulator. We record the interaction actions with the simulator during the operation process and count the operators and operands for each step to calculate the TEC value. Since the calculation results have different dimensions, we normalize the data, as shown in Table 6.

Prior to conducting factor analysis, we first conducted the KMO and Bartlett’s tests on the data. All statistical analyses were conducted using the SPSS data analysis software (version R27.0.1.0). The results of the data analysis are as follows: The KMO value is 0.844, and Bartlett’s sphericity test yields *p* < 0.001. A KMO value greater than 0.6 indicates that the data are suitable for factor analysis, and Bartlett’s sphericity test further confirms the data’s suitability. Through factor analysis, we extracted one main factor, with a rotated variance explanation rate of 78.995%, indicating that this factor has good explanatory power for the indicator data. In the factor loading coefficient matrix, all indicators have communality values higher than 0.4, indicating strong correlations between the indicators and the factor. We used linear combination coefficients to calculate the weights. First, we calculated the linear combination coefficients by dividing the loading coefficients by the square root of the corresponding eigenvalues. Next, we calculated the comprehensive score coefficients by multiplying the linear combination coefficients by the variance explanation rate and summing them, then dividing by the cumulative variance explanation rate. Finally, we normalized the comprehensive score coefficients to obtain the weight values for each indicator. After the calculations, the weights for TIC, TLC, TSC, and TEC are 25.87%, 23.27%, 25.54%, and 25.32%, respectively.

## 4. Experimental Validation

To validate the effectiveness of the measurement method, we hypothesize that task complexity is positively correlated with task execution time and workload. As task complexity increases, task execution time and workload should also increase. To test this, we compare the subjective scale evaluation results with the average execution time of the steps and analyze them in conjunction with the STC value. In the experiment, we recruited six participants, including three males and three females, who are all graduate students from our laboratory. All participants had accumulated simulator operation experiments through weekly experiments over the past 2–3 years, so they had some experience with simulator operations. Before the experiment, we provided detailed explanations of the experiment’s purpose, process, and precautions to ensure that the participants fully understood the experiment. The experiment was conducted in six sessions, with one participant performing the experiment in each session. After completing the tasks, each participant filled out the NASA-TLX scale to subjectively evaluate the workload. We also recorded the simulator operation logs and video data during the experiment to statistically analyze the operation time for each step. This approach allows us to more accurately measure the task execution time and workload, thereby validating the effectiveness of the task complexity measurement.

### 4.1. Comparison of Task Complexity and NASA-TLX Scores

Subjective evaluation techniques have developed significantly over the past few decades, with several methods proposed, most of which focus on workload assessment [36,37]. Here, we selected NASA-TLX as the subjective evaluation method. Hill [38] compared four subjective workload rating scales across four dimensions: sensitivity, operator acceptance, resource requirements, and special procedures. The authors found that NASA-TLX and the OW scale performed better in terms of sensitivity and operator acceptance, with NASA-TLX having the highest user acceptance. Additionally, NASA-TLX provides more detailed and diagnostic data.

The NASA-TLX scale measures workload across six dimensions: mental demand, physical demand, temporal demand, performance, effort, and frustration. The weighted average score across these dimensions is used to evaluate the participants’ workload. First, participants determine the relative importance of the six dimensions to assign weights to each dimension. Then, the weighted sum of the participant’s ratings for each dimension is calculated to obtain the workload score for each step. The average workload score for each step is calculated by averaging the scores of the six participants. Table 7 presents the average workload scores and task complexity values for each step.

First, we conducted an error analysis of the NASA-TLX scores, with the results shown in Table 8. We used SPSS software (version R27.0.1.0) to calculate the mean, standard deviation, and credibility of the results at the 95% confidence interval. The data in Table 8 show that the standard deviation of the NASA-TLX scores is 11.61, indicating some fluctuation around the mean but an overall relatively concentrated distribution. This may suggest that the factors affecting the score are relatively stable. In contrast, the standard deviation of the operation time reaches 34.49, a relatively large value, indicating a high degree of dispersion around the mean for operation time. However, the overall experimental data demonstrate a certain degree of acceptable consistency and stability.

To verify the reliability of the scores, we performed an Intraclass Correlation Coefficient (ICC) analysis [39]. The ICC is a statistical indicator used to measure data reliability and consistency. Typically, an ICC value greater than 0.75 indicates high consistency, a value between 0.40 and 0.75 indicates moderate consistency, and a value below 0.40 indicates poor consistency. The calculated ICC value is 0.804, indicating that the NASA-TLX scores are reliable.

**Figure 12:**
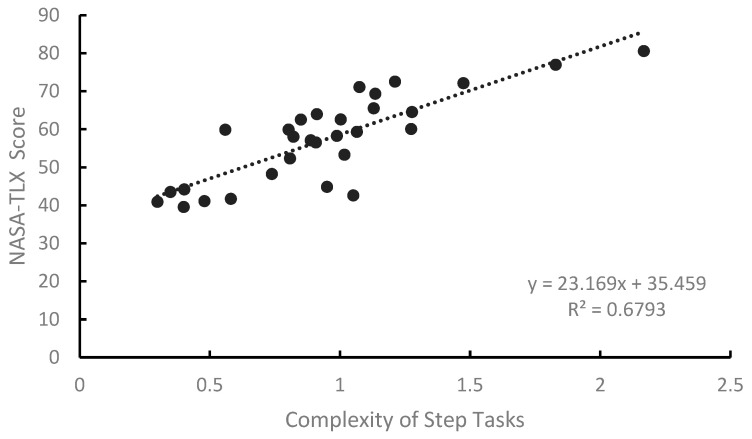
Linear regression graph of NASA-TLX scores and STC values.

We used SPSS (Version R27.0.1.0) to perform linear regression analysis on the STC values and NASA-TLX scores, as shown in Figure 12. Table 9 shows the analysis of variance (ANOVA) results. From Figure 12, we can see that task complexity is positively correlated with the NASA-TLX scores, with a coefficient of determination R^2^ = 0.679. The model has a good fit, and the regression coefficients are statistically significant (*p* < 0.001 *p* < 0.001). The regression equation is NASA-TLX Score = 23.160·STC+35.459. The ANOVA results show that the model is significant (F(1, 28) = 59.281, *p* < 0.001).

### 4.2. Comparison of Task Complexity and Operation Time

**Figure 13:**
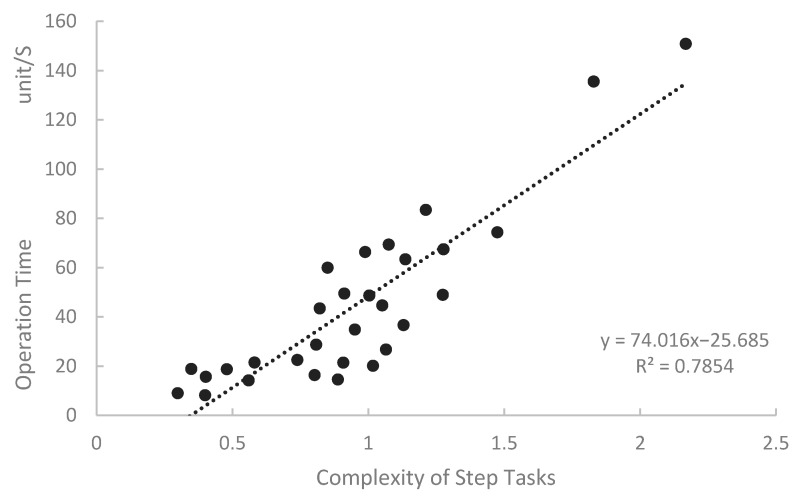
Linear regression graph of operation time and STC values.

We used the SLOCA emergency accident operating procedure, extracting 30 operation steps, mainly divided into reactor shutdown procedures and cooldown and depressurization procedures. We defined the time for each step as the period from when the operator starts reading the steps’ instructions to when all sub-tasks are completed. The final time is the average operation time of the six participants, as different participants have varying levels of experience, reaction speed, and knowledge. Table 10 shows the operation time and task complexity for each step. The regression analysis of the operation time and task complexity is shown in Figure 13, and the ANOVA results are shown in Table 11.

From Figure 13, we can see that the model has a good fit, with a coefficient of determination R = 0.785. Task complexity is significantly positively correlated with operation time, indicating that the task execution time is significantly influenced by task complexity. As task complexity increases, the task execution time also increases. The ANOVA results show that the model is significant F(1, 28) = 96.706, *p* < 0.001, and the regression coefficients are statistically significant (*p* = 0.004, *p* < 0.001). The regression equation is Operation Time = 74.016·STC-25.685.

### 4.3. Analysis and Discussion

In this study, we constructed a quantitative framework for measuring task complexity in human–computer interactions based on Halstead’s E measure and task entropy metrics. The validity of this framework, as well as the feasibility of complexity calculation, was verified through experiments. During the experimental phase, college students with an engineering background were recruited to participate in simulated process tests to preliminarily assess the explanatory power of the quantified results for operational performance indicators. From the experimental data, it is evident that the operation time and STC values exhibit a strong linear relationship (R^2^ = 0.785), indicating that task complexity has a direct and significant impact on execution time. The fit between NASA-TLX scores and STC values is not as good as that of the operation time. This may be because the STC values are objective measures of task complexity. Higher task complexity requires more operations and logical judgments, directly leading to longer operation times. Unlike subjective scores, the operation time is not influenced by individual perceptions. Therefore, we believe that the stronger fit between operation time and STC values is reasonable. However, it is important to note that the participants in this experiment were graduate students majoring in nuclear engineering whose professional experience is significantly lower than that of actual nuclear power plant operators. Studies have shown [40,41] that in human–computer interaction tasks with a high cognitive load, experience and professional knowledge are significant variables affecting decision-making speed and accuracy. Compared with experts, students have a lower level of experience and knowledge, so they allocate more cognitive resources to task processing when making decisions, resulting in greater delays between actions. Consequently, they may exhibit higher workload or error rates in some tasks. Therefore, there are errors in the actual task execution time and load ratings, which, in turn, affect the validity of the model.

Subjective scores reflect individuals’ perceptions of task complexity, which can be influenced by factors such as experience, skill level, and psychological state. Therefore, different individuals may perceive the same task as having different levels of complexity. For example, more experienced operators may require less detailed descriptions of operating procedures, while less experienced operators may prefer more detailed descriptions. For instance, in the task of opening spray valves for cooldown and depressurization, less experienced operators may expend cognitive resources to determine which system the spray valves belong to and what their symbols are. They may prefer descriptions such as “Open 001VP and 002VP for cooldown and depressurization.” Therefore, even steps with low STC values may impose a higher workload on less experienced operators. According to Rasmussen’s Skill–Rule–Knowledge (SRK) theory [42], when individuals are in a skill-based behavior state, their actions are highly automated, consuming fewer cognitive resources, such as continuously opening valves, starting pumps, or stopping pumps. However, when individuals face ambiguous scenarios or complex problems, they must rely on long-term memory for reasoning, entering a knowledge-based behavior state that requires significant cognitive resources. For example, in the step “Check if safety injection is needed; if needed, manually activate it; if not, execute procedure ES-0.1,” operators must make decisions based on the system’s status and their experience. Even if the step has low logical and information complexity, it may still impose a high cognitive load on operators.

The efficiency of operators in performing tasks, namely interacting with the interface, largely depends on the quality of the interface design. The total time for operators to perform interface tasks is the sum of information search time and operation execution time. A well-designed interface with effective information distribution and superior operation logic can significantly reduce the operator’s workload and interaction time. Complex interfaces and operation logic increase the information search time and the number of operation steps, leading to higher TEC values, longer task execution times, and an increased workload. Therefore, the quality of interface design is a major factor contributing to variations in operation time data.

## 5. Summary and Prospects

This study focuses on the complexity of human–computer interaction tasks in the digital control system (DCS) of nuclear power plants and proposes a multi-dimensional quantitative model that integrates dynamic interaction and static analysis. Through theoretical development, methodological improvements, and experimental validation, the mechanisms by which task complexity affects operational efficiency and cognitive load are revealed, and its practical application value in interface optimization is explored.

The core of the task complexity proposed in this study lies in the nonlinear coupling of static structure and dynamic interaction. As discussed in Section 2, traditional entropy measurement methods can effectively evaluate static complexity, but they are limited in quantifying dynamic interaction processes. For example, the task in step 25 involves five types of operators and ten types of operands. The cognitive load amplification caused by redundant interface navigation paths and operator reuse cannot be fully explained by static entropy values. To address this, Halstead’s E metric is introduced to dynamically quantify the interaction load between operators and operands. Experimental results demonstrate that the task execution complexity (TEC) value is significantly correlated with the task execution time, validating the independent contribution of dynamic interaction complexity.

From a practical perspective, this model provides a quantitative optimization pathway for the design of human–computer interfaces in nuclear power plants. For instance, high TEC values can be reduced by merging redundant operation windows, thereby improving operational efficiency and reducing the task execution time. Simultaneously, optimizing the entropy of information structure diagrams (TIC) can be achieved by modularizing information layouts, such as grouping nineteen types of nodes by function, thereby reducing the cognitive load. These measures align with Endsley’s [43] situation awareness theory, which emphasizes that simplifying information retrieval and operation paths enhances operators’ real-time perception of system states. Additionally, a task complexity grading based on task complexity (TC) values can guide targeted training design, such as increasing the simulation frequency for high-complexity steps or introducing decision flowchart aids.

However, this study has certain limitations. First, the experimental participants were trainees, and their skill levels may differ from those of professional nuclear power plant operators, potentially leading to prediction bias in the model. Future work could optimize the experiments by recruiting experienced operators to participate and compare their NASA-TLX scores and operation times with those of student participants. This could lead to the establishment of an experience-based correction factor for the TC values. Alternatively, complexity quantification tools could be embedded in routine training to analyze how the relationship between TC values and operational error rates evolves with increasing experience. Second, for future work, experimental optimization could be carried out by recruiting experienced operators for experiments to compare their NASA-TLX scores and operation times with those of student participants. This could lead to the establishment of an empirical correction coefficient for TC values. Alternatively, complexity quantification tools could be embedded in routine training to analyze the correlation between TC values and operation error rates and how this correlation evolves with increasing experience. At present, the complexity quantification framework mainly focuses on modeling the human-computer interaction process of a single operator and does not yet cover the “team complexity” factors under multi-role collaboration conditions. However, in the actual emergency operations of nuclear power plants, multiple roles need to collaborate to complete tasks within stringent time constraints, and the quality of information transfer, communication styles, and task load distribution among personnel have a profound impact on overall operational performance [44,45]. Therefore, in the future, a team performance model could be established, incorporating indicators such as the quality of team information transfer and the completeness of information sharing. Third, the weight coefficients are determined through static factor analysis and do not consider dynamic changes across task phases, such as the potential amplification of TEC effects under high-pressure conditions during early accident stages. Future work should validate the model’s robustness in real-world scenarios and explore the dynamic weight adjustment mechanisms, such as adaptive algorithms based on real-time workload feedback. Moreover, while this method has the potential to be extended to fields like aviation control and chemical systems, its generalizability requires verification through cross-domain experiments.

In summary, this study integrates Halstead’s E metric and entropy theory to construct a task complexity quantification framework that encompasses dynamic and static dimensions. Its theoretical value lies in improving the methodological system for complexity analysis, while its practical significance lies in providing actionable optimization tools for human factors engineering in nuclear power. Future work will focus on iterating the dynamic model, validating it across multiple scenarios, and expanding the research into team collaboration complexity to enhance the method’s applicability in high-risk industrial systems.

## Figures and Tables

**Table 1:** Definitions of interactive component actions.

Interaction Component	Abbreviation	Interactive Actions	Interactive Functionality Description
Valve	V	Click	Clicking the valve opens the operation window to perform related tasks
Pump	P	Click	Clicking the pump opens the operation window to perform related tasks
Controller	Ckg	Click	Clicking the controller opens the operation window to perform related tasks
	Cku	Click	
	Ckc	Click	
Navigation Icons	NI	Click	Click the navigation window icon to enter the system screen after powering on
Shortcut Navigation Icons	SNI	Click	The quick navigation icon enables fast screen transitions within the system with a single click
Execute Button	EB	Click	By clicking, confirm the execution of the operation
Exit Button	EXB	Click	Exit the window by clicking
Input Box	IB	Double-click	Select the window by double-clicking to enter data
Keyboard	Kbd	Input	Enter data by typing
Information Panels	IP	Check	Retrieve information by visual inspection
Operation Window	OW		A secondary window superimposed over the system interface for the operation of related components
System Window	SW		System screen
Reset Button	RB	Long press	Perform the reset operation of the related components by long-pressing
Switch Button	SB	Long press	Perform the on/off action of the related components by long-pressing
M/A Button	MAB	Click	Toggling between automatic and manual modes for the component
signal indicator light	SL	Check	“Determine system status by checking the signal lights.”

**Table 2:** First-order entropy classification of graphs.

Graph (a)			Class	Graph (b)		
In	Out	Node		In	Out	Node
0	2	a	I	0	2	a
1	1	b,c,d,e	II	1	1	b,d,e
2	2	d	III	1	2	c
2	0	g	IV	2	1	f
				2	0	g

**Table 3:** Second-order entropy classification of graphs.

Graph (a)		Class	Graph (b)	
Node	Neighbor Node		Node	Neighbor Node
a	b,c	I	a	b,c
b,c	a,d	II	b	a,g
d	b,c,e,f	III	c	a,d,c
e,f	d,g	IV	d,e	c,f
g	e,f	V	f	d,e,g
		VI	g	b,f

**Table 4:** Operation steps of some examples.

Action or Expected Response	Unintended Response
Step 23: Check if a train of safety injection pumps can be stopped	
a. Check the safety injection pumps-running	a. Execute Step 24
b. Determine the sub-cooling at the reactor core outlet required for pump shutdown according to Table XX	
c. Reactor core outlet sub-cooling-greater than the sub-cooling required by Table XX	c. Execute Step 27
d. Pressurizer water level-greater than 2.0 m	d. Do not stop the safety injection pump and return to Step 21
e. Stop a train of safety injection pumps	
f. Return to Step 23 a	
Step 24: Check if normal charging can be established	
a. Check the following items:	a. Execute Step 27
- All safety injection pumps are stopped	
- Centrifugal charging pump: one in operation and one on standby	
b. Reactor core outlet sub-cooling-greater than 20°C	b. If the reactor core outlet sub-cooling is less than 20 °C, start a high-pressure safety injection pump
c. Pressurizer water level-greater than 2 m	c. Return to Step 21
Step 25: Establish normal charging and maintain the pressurizer water level	
a. Place the RRA to RCV flow control valve in manual	
b. Check if the safety injection has been reset	b. Manually reset the safety injection
c. Manually fully open the charging valve	
d. Check the status of the following valves:	d. Restore the valves to the correct status
- Minimum flow valve of the charging pump-open	
- Charging line isolation valve-open	
- Boron injection isolation valve-closed	
e. Adjust the charging flow to maintain the pressurizer water level	

**Table 5:** Step 25 operation information.

Task Information	Operator Actions	Components and Information
1. Place the flow—control valve for RRA to RCV in manual mode.	1. Switch the valve to manual.	Valve: Data type B
2. Check if the safety injection has been reset.	2. Confirm that the safety-injection signal is cut off.	Signal indicator light: Data type B
3. Manually fully open the charging valve.	4.1 Set the charging valve to manual mode. 4.2 Adjust the charging-flow data.	Operating mode: Data type B Valve: Data type B
4. Check the status of the following valves.	4.1 Check that the minimum-flow pipeline isolation valve of the charging pump is in the open state. 4.2 Check that the charging-line isolation valve is in the open state. 4.3 Check that the boric-acid injection isolation valve is in the closed state.	Valve: Data type B Valve: Data type B Valve: Data type B
5. Adjust the charging flow to maintain the pressurizer water level.	5. Adjust the flow of the charging-flow valve and constantly monitor the pressurizer water level.	Charging flow: Data type P (process variable) Pressurizer water level: Data type P (process variable)

**Table 6:** Calculation results of step task complexity.

Step	TIC	TLC	TSC	TEC	Step	TIC	TLC	TSC	TEC
1	0.566	0.590	1.991	0.464	16	1.006	1.928	1.991	0.521
2	0.201	0.690	1.406	0.173	17	2.164	1.928	2.396	1.953
3	1.668	2.874	2.724	1.568	18	3.417	2.500	4.308	2.715
4	0.820	0.983	1.298	0.588	19	0.566	0.590	1.991	0.775
5	1.556	1.928	2.530	1.376	20	1.931	2.518	2.141	1.145
6	1.668	2.500	2.413	1.769	21	2.371	2.591	2.413	2.820
7	1.899	3.021	2.044	0.641	22	2.558	3.121	2.860	1.385
8	2.371	2.874	1.991	1.067	23	2.558	2.545	2.234	2.347
9	0.000	0.978	1.296	0.051	24	2.371	1.398	2.640	1.037
10	2.001	1.398	1.991	0.558	25	3.322	3.121	4.434	3.523
11	2.371	2.152	2.413	2.102	26	0.566	0.590	0.000	1.124
12	0.891	1.928	2.389	0.508	27	2.164	2.152	1.991	0.096
13	2.371	0.859	2.234	1.174	28	1.668	0.000	2.530	1.357
14	1.931	1.398	2.141	1.441	29	2.371	0.768	1.860	0.793
15	4.468	3.948	4.807	3.971	30	1.006	0.590	0.196	0.000

**Table 7:** NASA-TLX scores and task complexities of steps.

Step	NASA-TLX	STC	Step	NASA-TLX	STC
1	59.86	0.56	16	48.22	0.74
2	39.55	0.40	17	59.28	1.06
3	65.50	1.13	18	72.10	1.67
4	41.07	0.49	19	41.67	0.58
5	44.85	0.95	20	58.25	0.99
6	42.58	1.05	21	64.52	1.28
7	53.28	1.02	22	60.05	1.27
8	71.10	1.07	23	72.52	1.21
9	44.17	0.40	24	62.58	1.00
10	52.32	0.81	25	76.93	1.83
11	69.32	1.14	26	43.48	0.35
12	59.90	0.80	27	56.52	0.91
13	63.97	0.91	28	62.53	0.85
14	57.07	0.89	29	58.02	0.82
15	80.55	2.17	30	40.88	0.30

**Table 8:** Error analysis of NASA-TLX scores and operation time.

Index	Mean	Standard Deviation	Lower Limit of 95% Confidence Interval	Upper Limit of 95% Confidence Interval
NASA-TLX score	57.42	11.61	53.08	61.75
Operation time	44.47	34.48	31.59	57.35

**Table 9:** Analysis of variance results for NASA-TLX scores and STC.

Model	Degrees of Freedom	F	Significance	R	R^2^
Regression	1	59.281	<0.001	0.824	0.678
Residual	28				
Total	29				

**Table 10:** Operation time and task complexity of steps.

Step	Operation Time (unit:S)	STC	Step	Operation Time (unit:S)	STC
1	14.23	0.56	16	22.55	0.74
2	8.20	0.40	17	26.74	1.06
3	36.65	1.13	18	74.33	1.67
4	18.74	0.49	19	21.48	0.58
5	34.86	0.95	20	66.34	0.99
6	44.64	1.05	21	67.45	1.28
7	20.12	1.02	22	48.96	1.27
8	69.34	1.07	23	83.44	1.21
9	15.67	0.40	24	48.69	1.00
10	28.74	0.81	25	135.59	1.83
11	63.41	1.14	26	18.86	0.35
12	16.40	0.80	27	21.44	0.91
13	49.48	0.91	28	59.97	0.85
14	14.56	0.89	29	43.45	0.82
15	150.89	2.17	30	9.00	0.30

**Table 11:** Analysis of variance results for operation oime and STC.

Model	Degrees of Freedom	F	Significance	R	R^2^
Regression	1	96.706	<0.001	0.880	0.785
Residual	28				
Total	29				

## Data Availability

Data are contained within this article.

## Appendix A

**Table A1:** Example SLOCA Emergency Operating Procedure.

Step	Task Content
1	Confirm reactor trip
2	Check if the safety injection is activated
3	Confirm the isolation of feed-water
4	Confirm the operation of the auxiliary feed-water pump
5	Confirm the operation of the following equipment
6	Check whether the main steam pipeline should be isolated
7	Confirm that no containment spray is required
8	Confirm the safety injection flow rate
9	Confirm that the total flow rate of the auxiliary feed-water is normal
10	Check the pressurizer relief valve and spray valve
11	Check whether the main feed-water pump has stopped
12	Check whether the secondary side of the steam generator has a fault
13	Check whether the heat-transfer tubes of the steam generator are ruptured
14	Check the following pressures are normal
15	Check whether the secondary side pressure of the intact steam generator should be reduced to the system pressure
16	Check whether the main system is intact
17	Check the state of the pressurizer relief valve and its isolation valve
18	Check whether the safety injection can be terminated
19	Safety injection reset, containment ventilation isolation reset
20	Stop one high-pressure safety injection pump
21	Depressurize the main system to refill the pressurizer
22	Check whether one main pump should be started
23	Check whether one safety injection pump can be stopped
24	Check whether normal charging can be established
25	Establish normal charging and maintain the pressurizer water level
26	Check the operation status of the main pump
27	Confirm that no more safety injection flow is required
28	Check whether the safety injection tank should be isolated
29	Continuously cool and depressurize the main system
30	Continuously monitor the main system pressure and the pressurizer water level
